# Independent and joint associations between urinary polycyclic aromatic hydrocarbon metabolites and cognitive function in older adults in the United States

**DOI:** 10.3389/fpubh.2024.1392813

**Published:** 2024-08-07

**Authors:** Xin Lu, Yanan Zhou, Qingshan Miao, Xuexue Han, Yi Zhou, Gaofeng Zhao, Hao Yu, Min Chen

**Affiliations:** ^1^School of Mental Health, Jining Medical University, Jining, China; ^2^Daizhuang Hospital, Jining, Shandong, China

**Keywords:** polycyclic aromatic hydrocarbons, cognition impairment, older adults, NHANES, cross-sectional study

## Abstract

**Background:**

Polycyclic aromatic hydrocarbons (PAHs), as organic pollutants widely present in daily environments, have been shown by existing epidemiological studies to be significantly associated with deficits in learning and memory functions in children and adults. However, the association between exposure to PAHs and cognitive function in older adults remains unclear. Additionally, existing related studies have only assessed the association between individual PAH exposures and cognitive assessments, overlooking the risks posed by mixed exposures. This study aims to use three statistical models to investigate the individual and overall effects of mixed PAH exposures on the cognition of older adults in the United States.

**Methods:**

The study cohort was obtained from the NHANES database, which included individuals aged 60 and older from 2011 to 2014. Weighted generalized linear models (GLM), weighted quantile sum (WQS) models, and Bayesian kernel machine regression (BKMR) models were utilized to evaluate the connections between urinary PAH metabolites and the standardized Z-scores of four cognitive tests: Immediate Recall Test (IRT), Delayed Recall Test (DRT), Animal Fluency Test (AFT), and Digit Symbol Substitution Test (DSST).

**Results:**

Our analysis involved 899 individuals aged 60 and above. In the fully adjusted GLM, 2-hydroxynaphthalene (2-OHNa), 3-hydroxyfluorene (3-OHFlu), and 2-hydroxyfluorene (2-OHFlu) demonstrated negative associations with DSST Z-scores. In the WQS model, six urinary PAH metabolites were negatively linked to AFT Z-scores (β (95% confidence intervals [CI]): −0.120 (−0.208, −0.033), *p* = 0.007) and DSST Z-scores (β (95% CI): −0.182 (−0.262, −0.103), *p* < 0.001). In both assessments, 2-OHNa exerted the greatest influence among the urinary PAH metabolites. In the BKMR model, there was an overall negative correlation between urinary PAH metabolites and AFT and DSST Z-scores when the concentration was within the 25th to 75th percentile, where 2-OHNa dominated the main effect of the mixture. The WQS and BKMR models were adjusted for all covariates.

**Conclusion:**

Increased concentrations of urinary PAH metabolites are associated with cognitive decline in older adults, mainly on language ability, executive function, sustained attention, working memory, and information processing speed, with 2-OHNa playing a major effect.

## 1 Introduction

Polycyclic aromatic hydrocarbons (PAHs) are a type of organic compounds that contain multiple aromatic rings. Apart from natural emissions such as volcanic eruptions and forest fires, they can also be generated from incomplete combustion of fossil fuels, vehicle emissions, food cooking, and industrial production processes (1–3). As widely present organic pollutants in our daily environment (4), they can be absorbed by the human body through various pathways such as respiratory, digestive, and dermal contact (5, 6), resulting in various impacts on human health, including malignant tumors, respiratory system diseases, liver diseases, kidney diseases, and cardiovascular diseases (7–11).

In addition to their teratogenic, mutagenic, and carcinogenic effects, the association between PAH exposure and neurocognitive function has been studied across various populations. Research has shown that in infants and young children, prenatal exposure to higher levels of PAHs significantly compromises their cognitive development and language ability by the age of three. This exposure also contributes to anxiety, depression, and attention problems by the ages of six to seven (12, 13). Perera et al. (13) have shown that fetal and childhood exposure to environmental pollutants such as PAHs is linked to decreased intellectual development, increased behavioral problems, and abnormalities in brain structure and function. Furthermore, these pollutants may also contribute to neurobehavioral disorders in children, such as attention-deficit/hyperactivity disorder (ADHD) (14). Mortamais et al. (15) observed an association between high PAH exposure during childhood and reduced volume of the caudate nucleus, suggesting possible effects on brain structure and function through mechanisms like oxidative stress and receptor binding. In adults, Fu et al. (16) discovered a negative correlation between the levels of urinary PAH metabolites and the total score of the Montreal Cognitive Assessment (MoCA) as well as the score of visual–spatial/executive functions in populations with high PAH exposure, such as coke oven workers. Wang et al. (17) found an association between occupational exposure to PAHs and cognitive and neurobehavioral impairments in coal mine workers, manifested as slowed information processing speed and weakened auditory–visual memory. Du et al. (18) discovered that specific levels of PAH metabolites in the urine of coking plant workers were negatively correlated with cognitive test scores, and there was a dose–response relationship. Cho et al. (19, 20) also discovered a significant correlation between certain urinary PAH metabolites and reduced thickness of specific brain cortex in Korean adults. However, current research on the association between PAH exposure and cognitive function in the general old population in the United States is very limited. Moreover, the aforementioned studies utilized only single pollutant models, overlooking the substantial risks posed by the combined effects of mixed exposures in the environment.

This study employed general population data from the National Health and Nutrition Examination Survey (NHANES) in the United States to determine the correlation between various PAH exposures and cognitive function in the old population. It has been reported that the urinary levels of OH-PAHs can serve as biomarkers for assessing environmental exposure to PAHs (21). Furthermore, cognitive tests in the NHANES database were only available during the 2011–2012 and 2013–2014 cycles. Notably, during these periods, clear differences were observed in the types of urinary PAH metabolites among the surveyed populations. Given these limitations and to ensure a sufficiently large sample size, our study attempted to reflect the general exposure levels to PAHs among the majority of U.S. populations across various residential and living environments by including six common urinary PAH metabolites found in these cycles. The effects on cognitive functions, both singular and combined, were assessed through fitting generalized linear models (GLM), weighted quantile sum regression (WQS), and Bayesian kernel machine regression (BKMR) models. The results of this cross-sectional study can inform further longitudinal studies to explore the correlation between PAH exposure and neurocognition.

## 2 Methods

### 2.1 Study subjects

The research cohort was derived from the NHANES spanning 2011 to 2014. Conducted by the National Center for Health Statistics (NCHS), part of the Centers for Disease Control and Prevention, NHANES is a cross-sectional survey that targets the general, non-institutionalized population. The survey culled information regarding demographic profiles, socioeconomic positions, dietary patterns, and health histories via in-home interviews. In addition, physical examinations were performed on participants at specifically equipped centers, and biological specimens, such as blood and urine, were obtained for analysis. Informed consent was duly obtained from all subjects, and the NHANES protocol was approved by the Research Ethics Review Board of National Health Statistics. This investigation amalgamated data on demographics, clinical exam findings, laboratory results, and self-reported questionnaires across two NHANES cycles, 2011–2012 and 2013–2014. Individuals 60 years and above who completed cognitive performance evaluations were collected. Upon elimination of subjects with incomplete datasets concerning exposure variables (notably six urinary PAH metabolites) and key covariates (such as age, gender, ethnicity, body mass index [BMI], alcohol use, tobacco use, cholesterol levels, blood pressure status, diabetes, and sleep disorders), 899 participants were included for subsequent analysis.

### 2.2 Measurements of urinary PAH metabolites

Within the NHANES, one-third of participants aged 60 and above underwent assessment for urinary monohydroxy PAH metabolites across two successive survey periods. These metabolites are recognized as enduring indicators of exposure to PAHs through various routes. Expert personnel collected the urine specimens at mobile clinics, after which samples were promptly refrigerated at temperatures of −20°C or below before being dispatched to the National Center for Environmental Health for detailed examination. Gluconic acid or sulfated OH-PAH metabolites in urine undergo enzyme digestion, extraction, and derivatization. A variety of analytical techniques were applied in different cycles to detect PAH metabolites in urine. Concretely, for the 2011–2012 cycle, isotope dilution coupled with gas chromatography and tandem mass spectrometry (GC–MS/MS) was utilized. Conversely, for the cycle spanning 2013–2014, an online solid-phase extraction followed by high-performance liquid chromatography and tandem mass spectrometry (online SPE-HPLC-MS/MS) was utilized. It is worth noting that there are differences in PAH metabolites measured in urine between cycles. To reduce this difference, six PAH metabolites measured in all cycles were included in this study: 1-hydroxynaphthalene (1-OHNa), 2-hydroxynaphthalene (2-OHNa), 3-hydroxyfluorene (3-OHFlu), 2-hydroxyfluorene (2-OHFlu), 1-hydroxyphenanthrene (1-OHPh), and 1-hydroxypyrene (1-OHP).

### 2.3 Measurement of cognitive performance

Highly skilled medical professionals from the NCHS, a federal entity specializing in health data collection, administered cognitive evaluations at mobile examination facilities. These assessments aimed to gauge participants’ working memory, verbal fluency, and delayed recall. To ensure accuracy, participants permitted audio recording during the testing process, and each acknowledged their respective test scores.

During both the 2011–2012 and 2013–2014 NHANES cycles, cognitive function was evaluated using the Animal Fluency Test (AFT), Digit Symbol Substitution Test (DSST), and Consortium to Establish a Registry for Alzheimer’s Disease Word Learning (CERAD-WL) Test. These tests are known for their reliability in assessing cognitive abilities (22). We used the CERAD W-L sub-test to evaluate participants’ immediate and delayed memory for new vocabulary (23). This assessment included three consecutive immediate recall tests (IRT) and one delayed recall trial (DRT). In each IRT, participants read aloud 10 unrelated words, then immediately recalled as many as possible. The order of the words changes with each test, and the maximum score for a single test is 10 points. The DRT, conducted after the AFT and the DSST, aimed to assess long-term memory capacity for new vocabulary without cues, also with scoring from zero to 10. The AFT evaluates language abilities and executive functions by asking participants to list as many animal names as possible within 1 min, scoring one point per name (24). This test does not rely on formally educated experiences specific to any culture, making it applicable across diverse cultural backgrounds (25). Before the official AFT, participants were required to list three items of clothing in a practice test; failure in this practice disqualified participants from proceeding with the AFT. The DSST, derived from the Wechsler Adult Intelligence Scale (WAIS III), measures processing speed, sustained attention, and working memory (26). Participants had 2 min to match symbols to numbers according to a reference chart at the top of a paper form, filling 133 spaces with the corresponding symbols, with scores awarded for each correct match. A sample practice was conducted prior to the official test, disqualifying those who failed from continuing. It should be noted that there is no universally accepted benchmark for defining subpar cognitive performance across these four tests, and higher scores indicate enhanced cognitive abilities in all assessments.

### 2.4 Covariates

Apart from the aforementioned six urinary PAH metabolites, we examined various potential confounding factors, such as age, gender, race, BMI, alcohol consumption, smoking habits, high cholesterol, hypertension, diabetes, and sleep disorders. Age was treated as a continuous variable, while gender was categorized as male or female. Race and ethnicity encompassed Mexican Americans, non-Hispanic whites, non-Hispanic blacks, other Hispanics, and individuals of other races, including those of multiracial backgrounds. BMI was computed as weight divided by height squared and was also considered a continuous variable. Alcohol consumption status was determined based on consuming at least 12 alcoholic drinks within the past year. Smoking status was identified based on having smoked at least 100 cigarettes during one’s lifetime. Histories of high cholesterol, hypertension, diabetes, and sleep disorders were self-reported by participants following medical advice.

### 2.5 Statistical analysis

The cognitive scores were combined to obtain a total cognitive score, which was then grouped into quartiles for comparing participants’ demographic characteristics. Continuous variables were presented as mean and standard deviation (Mean ± SD) or median and interquartile range (M, IQR), while categorical variables were presented as counts (n) and percentages (%). Subsequently, descriptive statistics were performed for the six urinary polycyclic aromatic hydrocarbon metabolites, and Pearson correlation coefficients were calculated between them. Finally, three models were fitted by standardizing the four cognitive scores using Z-scores and log-transforming the six urinary polycyclic aromatic hydrocarbon metabolites.

#### 2.5.1 Generalized linear models

We utilized GLM to examine the association between individual urinary PAH metabolites and cognitive test scores. Two models were implemented: Model 1, which did not adjust for any covariates, and Model 2, which incorporated adjustments for covariates such as age, gender, race, BMI, alcohol consumption, smoking habits, high cholesterol, hypertension, diabetes, and sleep disorders. The effects of urinary PAHs were gauged through regression coefficients (Beta) alongside their corresponding 95% confidence intervals (CI).

#### 2.5.2 WQS regression model

The WQS regression model was applied to assess the collective impact of multiple urinary PAH metabolites on diverse cognitive test outcomes. This approach involved constructing weighted composite quantiles while controlling for covariates. Additionally, the model permitted an evaluation of each metabolite’s relative contribution to the overall WQS effect by estimating the weights of individual metabolites on the overall effect. In this study, the dataset was randomly partitioned into two subsets (40% for training and 60% for validation). Following 3,000 bootstraps, average empirical weights for each exposure factor were calculated.

#### 2.5.3 BKMR model

BKMR, a statistical method, estimates the individual and combined impacts of exposure mixtures by employing kernel functions to best represent these mixtures. This method presents a new approach for assessing the joint health effects of multiple exposures. Utilizing kernel functions enables flexible estimation of multivariable exposure-response functions, accommodating non-linear and non-additive effects while adjusting for covariates, including potential confounders. The method’s hierarchical variable selection addresses multicollinearity concerns by grouping highly correlated exposures and simultaneously selecting relevant exposure groups and individual exposures within each group. Furthermore, unlike the WQS model, BKMR allows for visualizing the response functions of individual exposures while considering other exposures and permits potential nonlinear relationships between exposure factors, as well as influences from different directions. For this study, the hierarchical variable selection method was iterated 10,000 times using the Markov chain Monte Carlo algorithm. Posterior inclusion probabilities (PIPs) were computed, and a threshold of 0.5 was utilized to determine the importance of urinary PAHs. All included covariates were adjusted in the BKMR model. Additionally, BKMR facilitates the evaluation of potential interactions between exposures. Detailed information regarding the estimates, visualization, and statistical aspects of BKMR analysis can be found in the study by Bobb et al. (27).

The data analysis was conducted utilizing R software version 4.2.2. The “gWQS” package was employed for WQS regression, while the “bkmr” package was used for BKMR. Two-sided *p*-values less than 0.05 were considered statistically significant.

## 3 Results

### 3.1 Population characteristics and correlations of PAH metabolites

**Figure 1:**
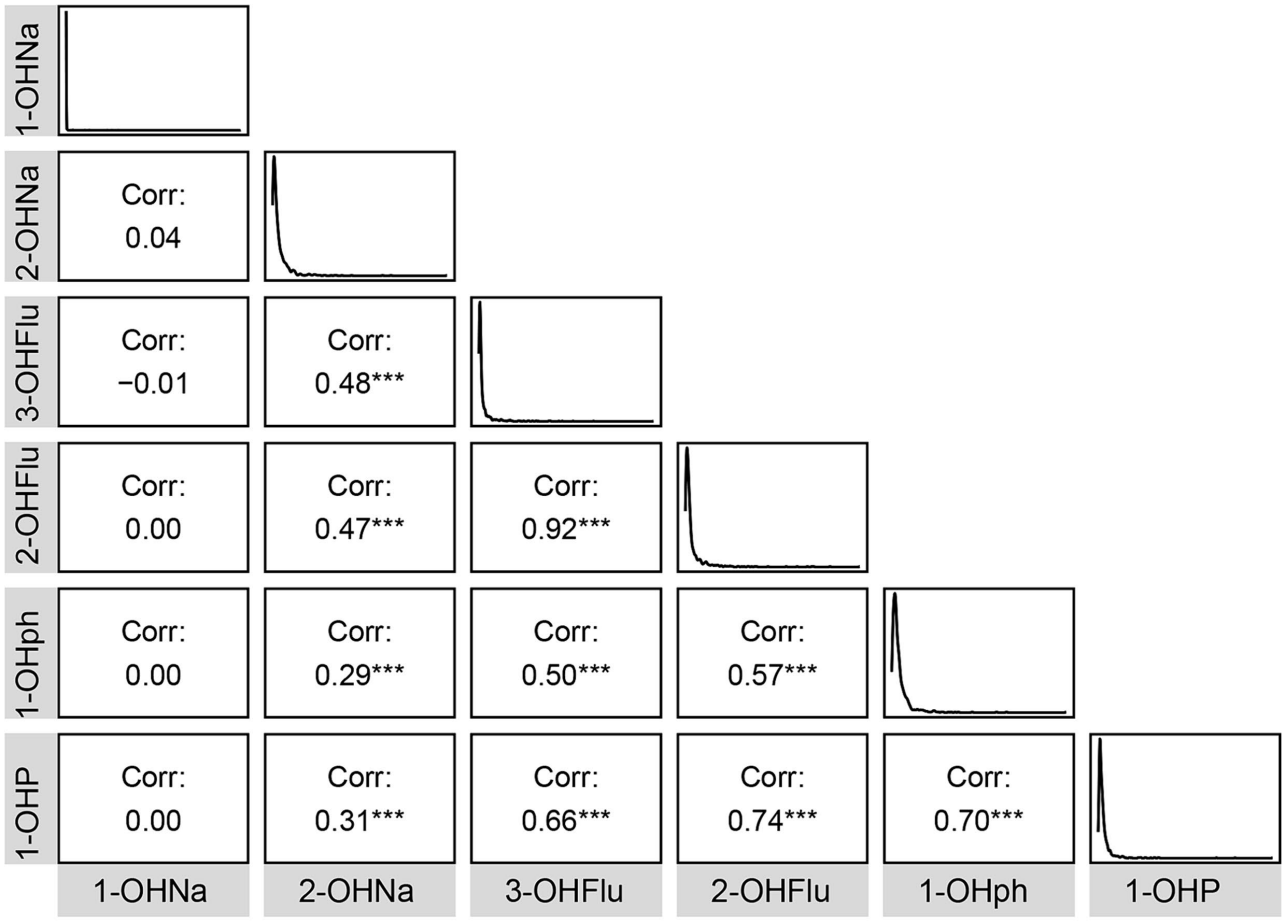
Pearson’s correlations among six urinary PAH metabolites. ^*^p < 0.05; ^**^p < 0.01; ^***^p < 0.001.

Table 1 displays the demographic profile of the participants. A total of 899 participants were included. The weighted median BMI was 27.7 (24.7, 31.9), and the weighted median age was 68 (63, 74) years. The cohort comprised 54% females (*n* = 455) and 46% males (*n* = 444). T majority of participants were non-Hispanic white (80%, *n* = 420), followed by non-Hispanic black (8.3%, *n* = 215). Most participants reported mild alcohol consumption (73%, *n* = 619), and over half were current smokers (53%, *n* = 462). Moreover, significant differences (*p* < 0.05) were observed between various cognitive score groups in age, gender, race, alcohol consumption, smoking habits, high blood pressure, and diabetes. Additionally, Figure 1 illustrates Pearson correlation coefficients between the six urinary PAH metabolites, revealing potential significant positive correlations between 1-OHP and 2-OHFlu, 1-OHP and 1-OHPh, and 2-OHFlu and 3-OHFlu.

**Table 1:** Weighted demographic characteristics of older adult Americans in different cognitive groups, NHANES, United States, 2011–2014.

Characteristic	*N* ^1^	Overall, *N* = 49,672,572^2^	Q1, *N* = 7,617,490^2^	Q2, *N* = 10,051,558^2^	Q3, *N* = 13,704,370^2^	Q4, *N* = 18,299,153^2^	*p*-value^3^
Alcohol drink	899						0.012
No		280 (27%)	77 (36%)	88 (35%)	70 (27%)	45 (18%)	
Yes		619 (73%)	151 (64%)	140 (65%)	157 (73%)	171 (82%)	
BMI	899						0.3
Median (IQR)		27.7 (24.7, 31.9)	27.8 (24.3, 32.0)	27.6 (24.3, 32.0)	28.9 (25.1, 33.1)	27.5 (24.8, 30.3)	
High blood pressure	899						0.027
No		347 (43%)	78 (32%)	79 (38%)	91 (43%)	99 (51%)	
Yes		552 (57%)	150 (68%)	149 (62%)	136 (57%)	117 (49%)	
High cholesterol level	899						0.6
No		384 (40%)	115 (48%)	94 (38%)	94 (41%)	81 (38%)	
Yes		515 (60%)	113 (52%)	134 (62%)	133 (59%)	135 (62%)	
Gender	899						0.023
Female		455 (54%)	90 (48%)	111 (48%)	118 (50%)	136 (62%)	
Male		444 (46%)	138 (52%)	117 (52%)	109 (50%)	80 (38%)	
Age	899						<0.001
Median (IQR)		68 (63, 74)	74 (68, 80)	71 (65, 77)	69 (65, 74)	64 (62, 70)	
Race/Ethnicity	899						<0.001
Mexican American		79 (3.4%)	35 (10%)	21 (4.2%)	17 (2.9%)	6 (0.6%)	
Non-Hispanic Black		215 (8.3%)	67 (15%)	63 (13%)	53 (7.5%)	32 (3.5%)	
Non-Hispanic White		420 (80%)	70 (59%)	92 (72%)	118 (83%)	140 (90%)	
Other Hispanic		89 (3.8%)	37 (10%)	26 (5.4%)	15 (2.3%)	11 (1.3%)	
Other Race - Including Multi-Racial		96 (4.4%)	19 (4.9%)	26 (5.6%)	24 (3.8%)	27 (4.1%)	
Diabetes	899						<0.001
Borderline		39 (4.5%)	9 (3.1%)	10 (4.6%)	11 (6.0%)	9 (3.8%)	
No		658 (77%)	144 (69%)	169 (70%)	163 (72%)	182 (89%)	
Yes		202 (18%)	75 (28%)	49 (25%)	53 (22%)	25 (7.4%)	
Sleep disorders	899						0.13
No		799 (88%)	202 (87%)	202 (86%)	195 (84%)	200 (92%)	
Yes		100 (12%)	26 (13%)	26 (14%)	32 (16%)	16 (8.0%)	
Smoking cigarettes	899						0.043
No		437 (47%)	100 (46%)	117 (41%)	106 (43%)	114 (55%)	
Yes		462 (53%)	128 (54%)	111 (59%)	121 (57%)	102 (45%)	

1, N not Missing (unweighted). 2, n (unweighted) (%). 3, Chi-squared test with Rao & Scott’s second-order correction; Wilcoxon rank-sum test for complex survey samples. IQR, interquartile range.

### 3.2 Association between PAH metabolites and cognitive test Z-scores using generalized linear model

As presented in Table 2, in the GLM without covariate adjustments, the levels of 1-OHNa, 2-OHNa, 3-OHFlu, and 2-OHFlu were significantly and negatively associated with DSST Z-scores. Furthermore, the level of 2-OHFlu was also negatively associated with IRT Z-scores. Upon adjusting for covariates only 2-OHNa, 3-OHFlu, and 2-OHFlu remained negatively associated with DSST Z-scores (Table 3).

**Table 2:** Association between individual urinary PAH metabolites and Z-standardized cognitive scores, crude model, NHANES, United States, 2011–2014.

Characteristic	IRT			DRT			AFT			DSST		
	Beta	95% CI^1^	*p*-value	Beta	95% CI^1^	*p*-value	Beta	95% CI^1^	*p*-value	Beta	95% CI^1^	*p*-value
1-OHNa	−0.037	−0.090, 0.017	0.171	−0.033	−0.084, 0.019	0.207	−0.049	−0.124, 0.027	0.199	−0.082	−0.140, −0.023	0.007
2-OHNa	−0.058	−0.130, 0.015	0.114	−0.023	−0.103, 0.056	0.555	−0.095	−0.195, 0.004	0.06	−0.139	−0.201, −0.078	<0.001
3-OHFlu	−0.045	−0.123, 0.033	0.245	−0.018	−0.087, 0.051	0.597	−0.039	−0.137, 0.059	0.418	−0.085	−0.142, −0.028	0.005
2-OHFlu	−0.09	−0.172, −0.009	0.03	−0.048	−0.125, 0.030	0.218	−0.069	−0.168, 0.031	0.169	−0.115	−0.180, −0.050	0.001
1-OHPh	−0.039	−0.146, 0.068	0.46	0.005	−0.105, 0.115	0.927	0.003	−0.116, 0.122	0.961	−0.014	−0.114, 0.085	0.769
1-OHP	0.008	−0.120, 0.135	0.904	0.085	−0.040, 0.210	0.176	−0.051	−0.170, 0.068	0.385	−0.06	−0.159, 0.039	0.225

1, CI = Confidence interval.

**Table 3:** Association between individual urinary PAH metabolites and Z-standardized cognitive scores, adjusted for all covariates, NHANES, United States, 2011–2014.

	IRT			DRT			AFT			DSST		
Characteristic	Beta	95% CI^1^	*p*-value	Beta	95% CI^1^	*p*-value	Beta	95% CI^1^	*p*-value	Beta	95% CI^1^	*p*-value
1-OHNa	−0.01	−0.055, 0.036	0.664	0.001	−0.045, 0.047	0.965	−0.027	−0.090, 0.036	0.381	−0.045	−0.096, 0.007	0.083
2-OHNa	−0.033	−0.096, 0.030	0.28	0.01	−0.057, 0.078	0.746	−0.079	−0.167, 0.008	0.073	−0.065	−0.121, −0.008	0.028
3-OHFlu	−0.041	−0.110, 0.028	0.225	0.003	−0.063, 0.070	0.917	−0.077	−0.177, 0.022	0.119	−0.073	−0.122, −0.024	0.006
2-OHFlu	−0.066	−0.143, 0.011	0.087	−0.009	−0.090, 0.071	0.806	−0.087	−0.186, 0.011	0.078	−0.071	−0.125, −0.016	0.014
1-OHPh	−0.008	−0.107, 0.092	0.869	0.043	−0.067, 0.153	0.422	−0.007	−0.119, 0.105	0.897	0.012	−0.072, 0.096	0.767
1-OHP	0.007	−0.114, 0.128	0.902	0.094	−0.025, 0.214	0.115	−0.106	−0.217, 0.004	0.058	−0.067	−0.165, 0.031	0.17

1, CI = confidence interval.

### 3.3 Association between PAH metabolites and cognitive test Z-scores using WQS regression model

**Figure 2:**
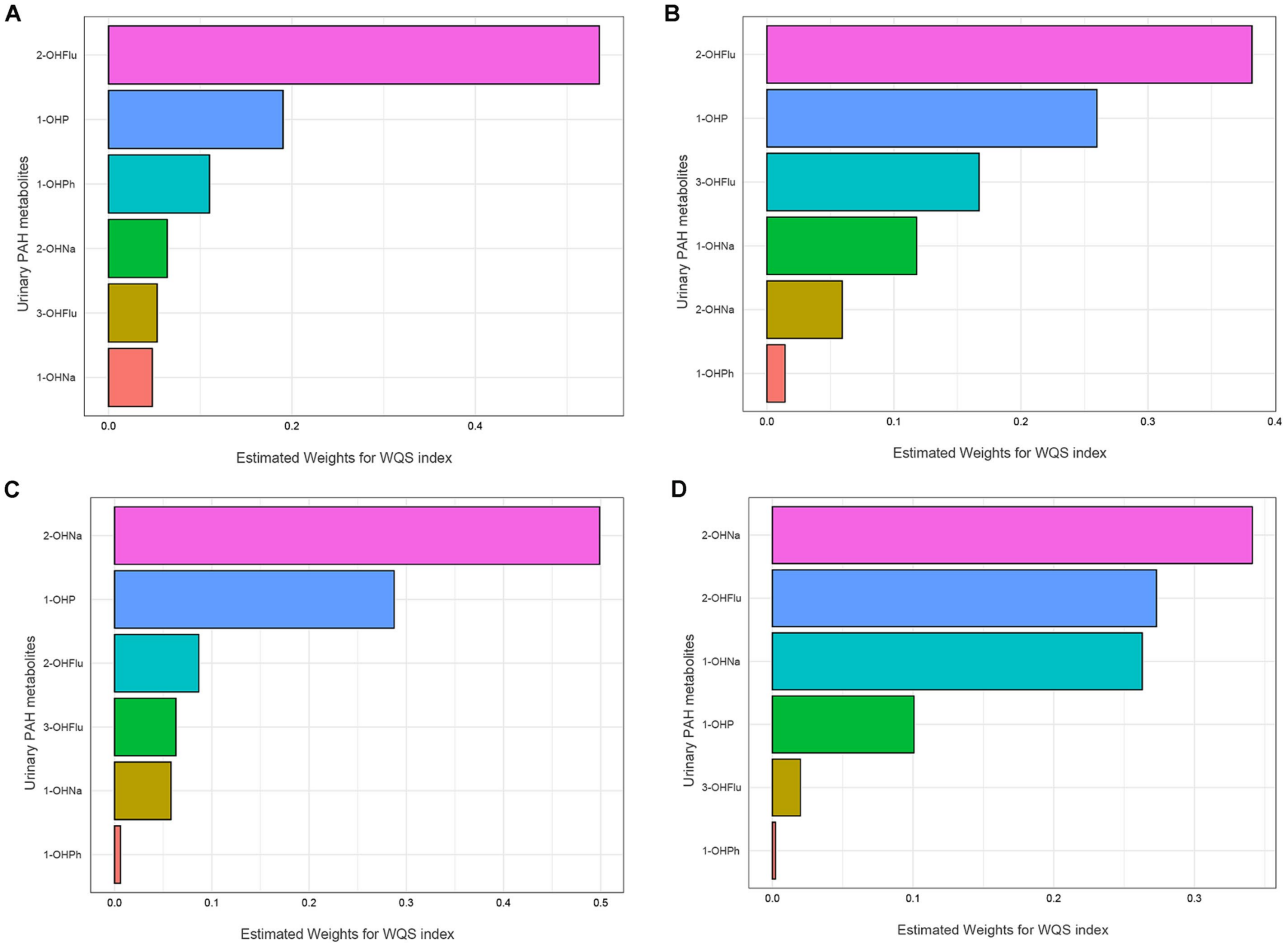
WQS model regression index weights for the IRT **(A)**, DRT **(B)**, AFT **(C)**, DSST **(D)**. Models were adjusted for all covariates. IRT, Immediate Recall test; DRT, Delayed Recall test; AFT, Animal Fluency test; DSST, Digit Symbol Substitution test.

The fully adjusted WQS regression model revealed a negative association of PAHs with AFT Z-scores [β (95% CI): −0.120 (−0.208, −0.033), *p* = 0.007] and DSST Z-scores [β (95% CI): −0.182 (−0.262, −0.103), *p* < 0.001], as detailed in Table 4. Figure 2 displays the relative weights of different urinary PAH metabolites for the four cognitive tests. 2-OHNa had the most predominant influence on both AFT and DSST.

**Table 4:** Association between WQS index and cognitive function, NHANES, United States, 2011–2014.

Outcomes	*β*	95% CI	*p*-value
IRT	−0.075	(−0.158, 0.007)	0.072
DRT	−0.029	(−0.115, 0.058)	0.514
AFT	−0.120	(−0.208, −0.033)	0.007
DSST	−0.182	(−0.262, −0.103)	<0.001

### 3.4 Association between PAH metabolites and cognitive test Z-scores using BKMR model

**Figure 3:**
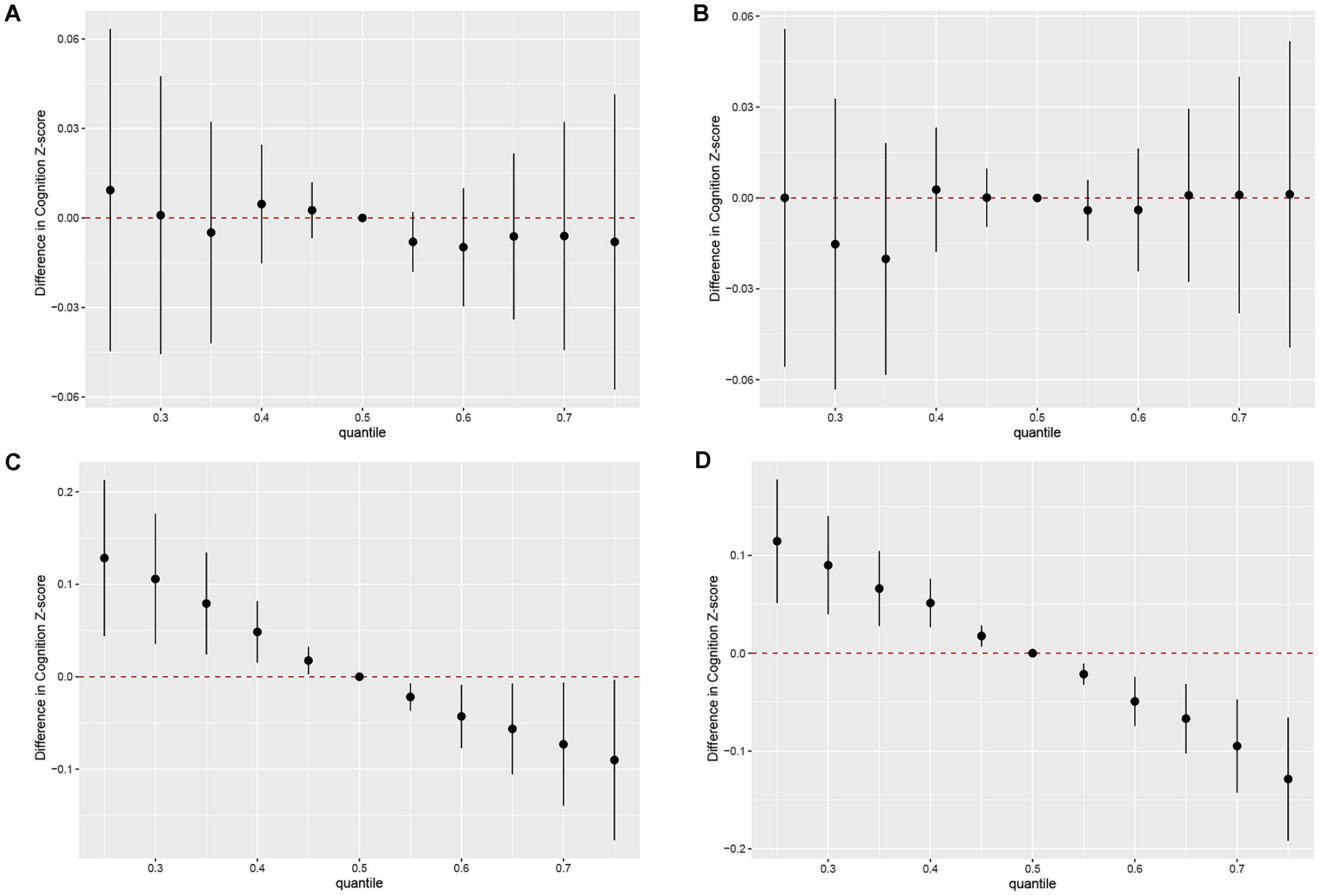
Joint effect of the mixture on z-scores of IRT **(A)**, DRT **(B)**, AFT **(C)**, DSST **(D)**. Models were adjusted for all covariates. Y-axis represents the estimated difference in z-scores when all urinary PAH metabolites were fixed at specific quantiles (ranging from 0.25 to 0.75), as compared to when urinary PAH metabolites were at the 50th percentile. Dots indicate the estimate, and black vertical lines represent 95% CIs. IRT, Immediate Recall test; DRT, Delayed Recall test; AFT, Animal Fluency test; DSST, Digit Symbol Substitution test.

As shown in Figure 3C, the concentrations of all urinary PAH metabolites within the 25th to 75th percentile range were negatively correlated with AFT Z-scores compared to the 50th percentile. In this negative correlation, 2-OHNa played the most significant role (cond PIP = 0.772) (Supplementary Table S1). Similarly, we found that the concentrations of the urinary PAH mixture within the 25th to 75th percentile range were more significantly negatively correlated with DSST Z-scores compared to the 50th percentile (Figure 3D). Once again, 2-OHNa dominated the overall negative effect of the mixture on cognitive scores (cond PIP = 0.999). However, for the DRT and IRT models, no clear trends were observed as the 95% CIs of all estimated overall effects included 0, indicating no significant association between the overall mixture concentration changes and cognitive scores (Figures 3A,B).

**Figure 4:**
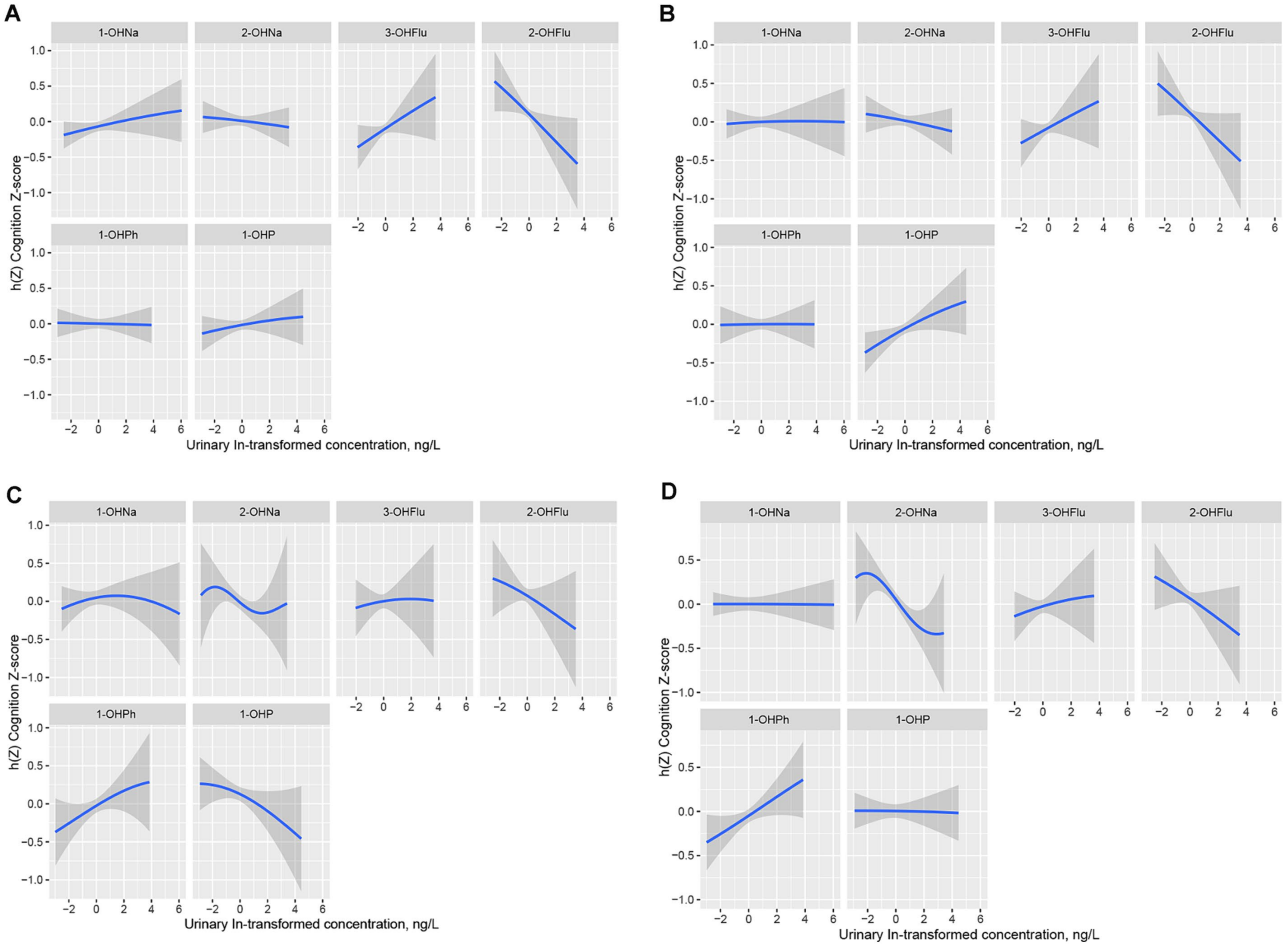
Univariate exposure-response functions (95% CI) in IRT **(A)**, DRT **(B)**, AFT **(C)**, DSST **(D)** for single urinary PAH metabolite when other five urinary PAH metabolites fixed at the median. Models were adjusted for all covariates, Y-axis represents the estimated difference in z-scores at a given level of urinary PAH metabolite compared to its median level when the other five urinary PAH metabolites were all at their median. h(Z) can be interpreted as the relationships between urinary PAH metabolites (ln, ug/L) and the Cognition Z-score. IRT, Immediate Recall test; DRT, Delayed Recall test; AFT, Animal Fluency test; DSST, Digit Symbol Substitution test.

Figure 4 displays the univariate exposure-response function trends of each urinary PAH metabolite with the four cognitive test scores when the other metabolites are fixed at their 50th percentile. We specifically focused on the significant impact of the mixture concentration on cognitive test scores in AFT and DSST. In AFT, 2-OHNa, 2-OHFlu, and 1-OHP showed a decreasing association with AFT Z-scores over the range from −2 to 2 on the x-axis (Figure 4C), with these three urinary PAH metabolites having the highest cond PIPs in the AFT test. Meanwhile, in DSST, only 2-OHNa and 2-OHFlu showed a weakening association with DSST Z-scores (Figure 4D).

Finally, we further investigated the interactions between the six urinary PAH metabolites in the four cognitive tests. By fixing the concentrations of the other metabolites at their median levels, the concentration of the second urinary PAH metabolites was fixed at its 25th, 50th, and 75th percentiles, and the exposure-response functions of each urinary PAH metabolite under different concentrations of the second metabolites were determined (Supplementary Figure S1). We found that, among the four cognitive tests, only in AFT, 2-OHNa exhibited a certain interaction with 2-OHFlu, while there were no apparent interactions among the other exposure factors in AFT and among all exposure factors across the other cognitive tests, as the slopes of the bivariate response functions were similar at different percentiles of the second urinary PAH metabolites, or the curves were almost parallel.

## 4 Discussion

In this cross-sectional study involving 899 older adults, three statistical models were used to further analyze the association between PAH exposure and cognitive function. In the GLM, after adjusting for all covariates, we found that 2-OHNa, 3-OHFlu, and 2-OHFlu were negatively correlated with DSST Z-scores. In the fully adjusted WQS and BKMR models, we found that the overall effects of the six urinary PAH mixtures were negatively correlated with AFT Z-scores and DSST Z-scores, and in both cognitive tests, 2-OHNa was the predominant driver of the main effect of the mixture. Building on this, BKMR also revealed a potential interaction between 2-OHNa and 2-OHFlu in the AFT cognitive test. In this study, scores from the AFT primarily reflected language capabilities and executive functions; scores from the DSST were indicative of sustained attention, working memory, and information processing speeds. Therefore, increased concentrations of urinary PAH metabolites are associated with cognitive decline in older adults, mainly on language ability, executive function, sustained attention, working memory, and information processing speed, with 2-OHNa playing a major effect.

In the independent factor analysis of PAHs, the results of Du et al.’s study were consistent with our findings. The team’s research on populations with occupational exposure, including coking plant workers, also revealed a negative correlation between 2-OHNa and specific cognitive test scores, alongside a dose–response relationship (18). Additionally, Jaelim Cho’s cohort study on Korean adults found a significant association between increased concentrations of 2-OHFlu and 1-OHP in urine and reduced thickness of the entire brain cortex, specifically increasing atrophy scores in Alzheimer’s disease (AD)-specific brain regions, including the frontal lobe, parietal lobe, temporal lobe, and cingulate gyrus (20). Another cross-sectional study by Jaelim Cho’s team showed a significant decrease in verbal learning and memory scores with increasing percentiles of 1-OHP concentration in adult urine (19). However, Jaelim Cho’s two studies only included the four common urinary PAH metabolites found in Asian countries, excluding 2-OHNa and 3-OHFlu from our study, and all the aforementioned studies overlooked the comprehensive effects of PAH mixtures on neurocognition.

Although the potential mechanisms underlying the association between PAH exposure and cognitive decline remain unclear, several plausible explanations for this association have been proposed. Existing research has shown that PAHs are lipophilic compounds that tend to accumulate in neural tissues and exert neurotoxicity (28). Furthermore, Saunders et al.’s study found that PAH exposure may induce oxidative stress (29), leading to increased reactive oxygen species levels and decreased antioxidant enzyme levels, potentially damaging DNA (30–32). Therefore, the direct cause of PAHs-induced neurotoxic effects may be related to oxidative stress. Cognitive alterations resulting from PAHs neurotoxicity may stem from disruptions in diverse brain neurotransmitter expression levels, with distinct microRNA expression also thought to be pivotal in PAHs-induced neurotoxic mechanisms (33). Furthermore, the connection between PAH exposure and cognitive changes may be closely associated with neurodegeneration. Several studies indicated a correlation between PAH exposure and cortical thinning in the brain (19, 20), considered indicative of neurodegenerative changes, particularly in critical regions such as the frontal, parietal, and temporal lobes that are pivotal for cognitive functions encompassing working memory, attention, auditory memory, language learning, and memory (34). Fu et al. (16) identified a positive correlation between urine PAH metabolite levels and plasma p-tau231 levels among populations with elevated PAH exposure, including coke oven workers. Multiple studies have considered the change in concentration of abnormal p-tau231 to reflect the progression of certain neurodegenerative diseases (35, 36). Additionally, the correlation between plasma p-tau231 and cerebrospinal fluid p-tau231 has been recognized as a diagnostic biomarker for AD (37, 38). This suggests that PAH exposure may accelerate or lead to the development of neurodegenerative changes by affecting the phosphorylation process of tau proteins. Partial experimental studies have also supported the impact of PAH exposure on neurodegenerative changes. Animal studies have demonstrated that PAHs affect neuropathology, including the down-regulation of antioxidant enzymes (29) and the up-regulation of neuronal apoptosis (39). A recent study indicated that PAHs (contained in particulate matter) via nasal exposure can upregulate inflammatory molecules (cytochrome P450 1A1, tumor necrosis factor-alpha, and cyclooxygenase-2) in the mouse brain through binding to aromatic receptors (40). This implied that neurodegenerative changes in the human body may also be related to oxidative stress and chronic neuroinflammation induced by PAH exposure. In summary, PAH exposure may lead to neurotoxicity and neurodegenerative changes through the induction of systemic inflammation and oxidative stress. The interplay of neurotoxicity and neurodegenerative changes may also have complex interactions, potentially impacting neurocognitive function. These processes involve various biological and molecular mechanisms, demonstrating the complexity of the impact of PAHs on human health.

In this study, 2-OHNa was the major contributor to the association of urinary PAH metabolites with cognitive decline in older Americans. In previous studies, 2-OHNa was one of the most frequently detected urinary PAH metabolites other than 1-OHP, and its urinary concentration was much higher than that of 1-OHP, reflecting PAH exposure in almost all settings (41). Among occupational exposures, research evidence suggests that occupationally exposed populations, such as coke oven workers, have significantly elevated urinary 2-OHNa concentrations relative to non-occupationally exposed populations (16, 42). In addition, urinary 2-OHNa concentrations appear to be higher in populations frequently exposed to combustion by agricultural burning or indoor solid fuels such as coal and wood (43, 44) relative to public emissions in the general community from automobile exhaust. Finally, chronic exposure to environmental tobacco and smoke such as smokers may also lead to a significant increase in urinary 2-OHNa concentrations (45). Based on studies of PAH metabolites such as 2-OHNa, there is a need to implement long-term and real-time PAH monitoring for these high-risk environments and populations. On the other hand, strict emission standards should be formulated to limit various activities that may release PAHs, such as industrial production, transportation, and agricultural incineration, and new technologies and clean energy sources should be promoted and applied, especially in high-risk areas, to reduce the generation and emission of PAHs.

The strengths and limitations of this study are worth noting. To the best of our knowledge, this is the first attempt to explore the association between urinary PAH metabolites and cognitive risk in the older adult population through a large-scale epidemiological study conducted in the general population of the United States. The results of this study are quite representative. Furthermore, the study conducted an in-depth analysis of the association between PAH exposure and cognitive changes, overcoming the traditional limitation of epidemiological research focusing solely on exposure to a single chemical substance. We employed diverse methodologies to evaluate and examine the individual and collective impacts of PAH exposure, complementing one another and enhancing the clarity and comprehensibility in explaining both the singular and combined effects of PAH exposure, as well as the contribution of each component. Nevertheless, this study has limitations. As a cross-sectional study, it is unable to establish the causal relationship between urinary PAH metabolites and changes in cognitive function. Secondly, owing to the limitations of the survey period and data processing, the inclusion of related types of urine PAHs is not comprehensive enough. In actual environments, PAHs can involve more than six different types. Lastly, some included covariates, such as self-reported medical history of diabetes, hypertension, and hypercholesterolemia, may not be accurate enough and could be subject to recall bias. Consequently, we strongly recommend that subsequent research should be longitudinal, prospective cohort studies, incorporating a sufficient variety of urinary PAH metabolites for long-term tracking to observe the dynamic effects of PAHs mixture exposures on neurocognitive functions over time. This approach could substantially reduce recall and confounding biases, clarifying causality to a greater extent.

## 5 Conclusion

Our study concludes that increased concentrations of urinary PAH metabolites in older Americans aged 60 years and older are associated with cognitive decline, mainly on language ability, executive function, sustained attention, working memory, and information processing speed, with the metabolite 2-OHNa playing a major effect. Given the potential additional health implications of PAHs, further comprehensive research and real-world monitoring of PAHs could aid in better mitigating the risks associated with PAH exposure. Additionally, more longitudinal epidemiological studies are warranted to confirm the correlation between PAHs and neurocognition.

## Data availability statement

The original contributions presented in the study are included in the article/Supplementary material, further inquiries can be directed to the corresponding author.

## Author contributions

XL: Funding acquisition, Conceptualization, Formal analysis, Investigation, Methodology, Writing – original draft, Writing – review & editing. YaZ: Conceptualization, Methodology, Writing – original draft. QM: Conceptualization, Writing – original draft, Formal analysis, Investigation. XH: Methodology, Writing – review & editing. YiZ: Methodology, Writing – review & editing. GZ: Methodology, Writing – original draft. HY: Funding acquisition, Investigation, Writing – review & editing. MC: Funding acquisition, Resources, Supervision, Writing – original draft.

## Supplementary material

SUPPLEMENTARY TABLE S1 Cond PIPs of urinary PAH metabolites in four cognitive tests within the BKMR model.
