# Supplementary material for: Independent and joint associations between urinary polycyclic aromatic hydrocarbon metabolites and cognitive function in older adults in the United States
Source: Front Public Health. 2024 Aug 7;12:1392813. doi: 10.3389/fpubh.2024.1392813 (PMC11335504; doi:10.3389/fpubh.2024.1392813)
Supplement: SUPPLEMENTARY TABLE S1 — Cond PIPs of urinary PAH metabolites in four cognitive tests within the BKMR model. [file Table_1.DOCX]

| IRT | Urinary PAH metabolite | condPIP |
| --- | --- | --- |
| 1 | 1-OHNa | 0.0242 |
| 2 | 2-OHNa | 0.0250 |
| 3 | 3-OHFlu | 0.0780 |
| 4 | 2-OHFlu | 0.1114 |
| 5 | 1-OHPh | 0.0068 |
| 6 | 1-OHP | 0.0398 |

| CRT | Urinary PAH metabolite | condPIP |
| --- | --- | --- |
| 1 | 1-OHNa | 0.0288 |
| 2 | 2-OHNa | 0.0430 |
| 3 | 3-OHFlu | 0.0596 |
| 4 | 2-OHFlu | 0.1062 |
| 5 | 1-OHPh | 0.0162 |
| 6 | 1-OHP | 0.0832 |

| AFT | Urinary PAH metabolite | condPIP |
| --- | --- | --- |
| 1 | 1-OHNa | 0.1540 |
| 2 | 2-OHNa | 0.7716 |
| 3 | 3-OHFlu | 0.1674 |
| 4 | 2-OHFlu | 0.2372 |
| 5 | 1-OHPh | 0.2086 |
| 6 | 1-OHP | 0.2284 |

| DSST | Urinary PAH metabolite | condPIP |
| --- | --- | --- |
| 1 | 1-OHNa | 0.0050 |
| 2 | 2-OHNa | 0.9986 |
| 3 | 3-OHFlu | 0.0690 |
| 4 | 2-OHFlu | 0.0740 |
| 5 | 1-OHPh | 0.1704 |
| 6 | 1-OHP | 0.0132 |
