# Supplementary material for: Independent and joint associations between urinary polycyclic aromatic hydrocarbon metabolites and cognitive function in older adults in the United States
Source: Front Public Health. 2024 Aug 7;12:1392813. doi: 10.3389/fpubh.2024.1392813 (PMC11335504; doi:10.3389/fpubh.2024.1392813)

Figure S1: Bivariate exposure-response functions for urinary PAH metabolites and four cognitive scores when another urinary PAH metabolite was fixed at varying (25th, 50th, 75th) percentiles and other urinary PAH metabolites were fixed at the median in the BKMR model.

Models were adjusted for all covariates. h (Z) can be interpreted as the relationships between urinary PAH metabolites (ln, ug/L) and the Cognition Z-score(A-IRT,B-DRT,C-AFT,D-DSST). IRT, Immediate Recall test; DRT, Delayed Recall test; AFT, Animal Fluency test; DSST, Digit Symbol Substitution test.


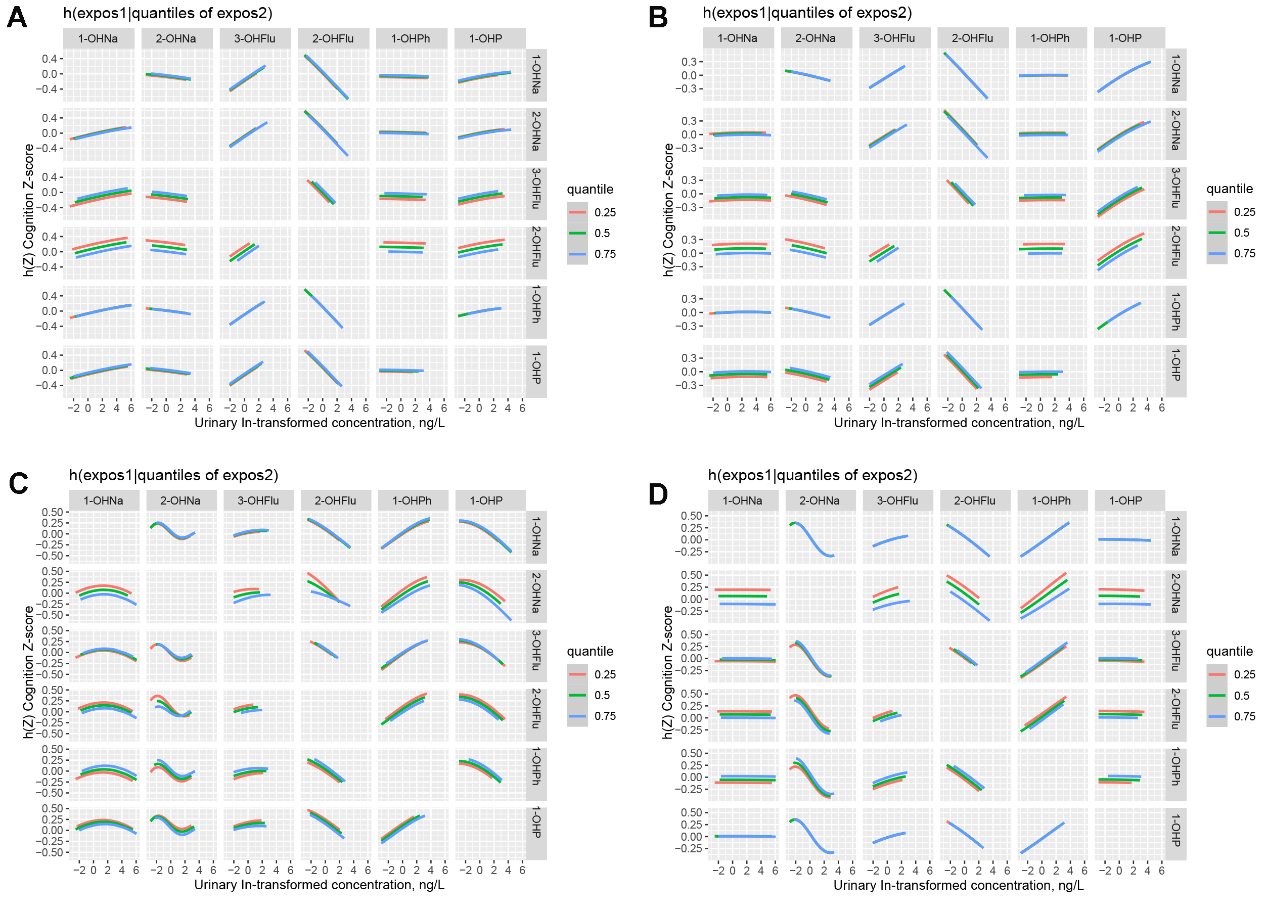

Supplement: Supplementary file 2 [file Data_Sheet_1.docx]
